# Cost-effectiveness analysis of GnRH-agonist long-protocol and GnRH-antagonist protocol for *in vitro* fertilization

**DOI:** 10.1038/s41598-020-65558-0

**Published:** 2020-05-26

**Authors:** Miaomiao Jing, Chenxi Lin, Wenjun Zhu, Xiaoyu Tu, Qi Chen, Xiufang Wang, Youbing Zheng, Runju Zhang

**Affiliations:** 1 Key Laboratory of Reproductive Genetics (Ministry of Education), Department of Reproductive Endocrinology, Women’s Hospital, Zhejiang University School of Medicine, Hangzhou, Zhejiang China; 2 Medical Quality Management Section, Women’s Hospital, Zhejiang University School of Medicine, Hangzhou, Zhejiang China; 3 Hangzhou Fuyang Women and Children Hospital, Hangzhou, Zhejiang China; 4 Zhejiang Xiaoshan Hospital, Hangzhou, Zhejiang China; 5 Shengzhou Maternal and Child Health Hospital, Shaoxing, Zhejiang China

**Keywords:** Health care, Health care economics

## Abstract

The gonadotropin releasing hormone agonist (GnRH-a) long-protocols and the GnRH-antagonist protocols are two commonly used protocols for *in vitro* fertilization (IVF), but their cost-effectiveness has not been studied, especially in China. A retrospective study involving 1638 individuals in GnRH-a long-protocol and 621 in GnRH-antagonist protocol were conducted and a decision tree model analysis was used to analyze the cost-effectiveness. Both direct and indirect costs were calculated. As a result, during the fresh embryo transplantation cycles, there was no significant difference in the rate of ongoing pregnancy between the two protocols, the average cost of per ongoing pregnancy in the GnRH-antagonist protocol was $ 16970.85, and that in the GnRH-agonist long-protocol was $19902.24. The probability of cumulative ongoing pregnancy per start cycle was estimated at 60.65% for the GnRH-antagonist protocol and 71.6% for the GnRH-agonist long-protocol (P < 0.01). Considering the cumulative ongoing pregnancy rate, the mean costs per ongoing pregnancy were estimated at $8176.76 and at $7595.28 with GnRH-antagonist protocol and GnRH-agonist long protocol, respectively. In conclusion, in fresh embryo transplantation cycle, the GnRH-antagonist protocol has economic advantage. However, the GnRH-agonist long protocol is more cost effective considering the cumulative ongoing pregnancy rate in the fresh embryo and frozen embryo transplantation cycles.

## Introduction

According to a World Health Organization (WHO) report, about 48.5 million couples are affected by infertility worldwide in 2010^1^. With the refinement and development of assisted reproductive technology (ART), increasing number of infertile couples seek ART. The European Society of Human Reproduction and Embryology (ESHRE) reported that the world-wide number of babies born as a result of ART has reached an estimated total of 8 million since the world’s first, Louise Brown, was born in July 1978^2^. Up to now, ART is the most important method to treat infertility in the world.

Study has shown that cumulative live birth rates are increased with the number of oocytes obtained^3^. Therefore, controlled ovarian hyperstimulation (COH) is an important process to obtain a set number of oocytes for IVF. The GnRH-agonists were introduced into IVF in the late 1980s and the GnRH-agonist long-protocol is still the most frequently used protocol in most centers worldwide^4^. The basic principle is to use gonadotropin-releasing hormone agonist (GnRH-a) to regulate pituitary and stimulate follicular growth with exogenous gonadotropin hormone, and avoid endogenous luteinizing hormone (LH) surge before oocyte retrieval^5,6^. Since 1990s, GnRH antagonists were used in COH, this protocol competitively blocks pituitary GnRH receptors, inducing a rapid, reversible suppression of gonadotrophin secretion and preventing and interrupting LH surges^6–8^. The GnRH-antagonist protocol have been widely adopted in IVF due to these advantages.

IVF is a protracted and costly process. In most developed countries, IVF is covered by insurance or subsidized. This is not the case in developing countries^9^. In China, the cost of IVF is on patients. High costs discourage low-income infertility couples from seeking ART treatment.

While both protocols are commonly used today, little is known about their cost-effectiveness, especially in China. Studies have shown that hormonal stimulation covered the main part of the costs per cycle^10^. Moolenaar *et al*. reported that most economic studies about ART were performed in countries from mainland Europe (38%) and the United States (34%)^11^. In China, there has been few economic researches on COH protocols. Herein, we performed a retrospective study on comparing the cost-effectiveness of the two protocols using a single center data in China. Choosing a cost-effective protocol, can not only ease the financial pressure on couples, but also provide reference for medical decision-making.

## Methods

### Patients

Individuals who came to the Reproductive Center of Women’s Hospital, Zhejiang University School of Medicine for their first cycle of IVF treatment from 1 January 2015 to 31 December 2017 were included. Inclusion criteria were as follows: 20 < age ≤ 38 years, regular ovulatory cycles every 21–35 days, total antral follicle count (AFC) ≥ 5, first cycle of IVF treatment, COH planned using the GnRH-agonist long-protocol or the GnRH-antagonist protocol, IVF fertilization. Exclusion criteria were the use of donor oocytes or frozen-thawed oocytes for fertilization, other protocols for COH, ICSI fertilization. Patient demographics are presented in Table 1.

**Table 1:** Patient demographics and infertility treatment-related characteristics.

Characteristics	GnRH-agonist long-protocol	GnRH-antagonist protocol	P value
Age(year)	29.26 ± 3.36	29.54 ± 3.32	0.078
Duration of infertility (year)	3.12 ± 2.34	3.28 ± 2.51	0.148
Height (cm)	159.57 ± 6.02	159.56 ± 4.60	0.964
Weight (kg)	55.52 ± 7.57	55.89 ± 7.26	0.305
BMI (kg/m^2^)	21.76 ± 2.81	21.95 ± 2.69	0.144
AFC (n)	14.52 ± 4.06	14.38 ± 4.30	0.463
bFSH (IU/L)	6.22 ± 1.67	6.73 ± 1.79	<0.01
bLH (IU/L)	5.67 ± 3.18	5.58 ± 2.99	0.52
bE2 (pmol/l)	116.67 ± 67.72	115.40 ± 64.83	0.688
Duration of Gonadotropin stimulation (day)	10.82 ± 1.78	9.53 ± 1.87	<0.01
Gonadotropin used (IU)	2021.09 ± 668.56	1823.78 ± 561.22	<0.01
Number of oocytes obtained (n)	13.90 ± 6.58	11.89 ± 6.70	<0.01
Number of embryos available (n)	4.42 ± 3.43	3.98 ± 3.27	0.005
Number of frozen embryos (n)	3.72 ± 3.74	3.20 ± 3.57	0.005
Average number of embryos transferred (n)	1.810 ± 0.40	1.79 ± 0.42	0.349
Number of Frozen embryo transplantable cycle (n)	2.05	1.78	—
Clinical pregnancy rate in the fresh embryo transplantation cycle	47.26%	49.44%	0.487
Ongoing pregnancy rate of fresh embryo transplantation	38.77%	37.22%	0.613
Cost in fresh embryo transplantation($)	19902.24	16970.85	—
Cumulative ongoing pregnancy rate	71.6%	60.65%	<0.01
Cumulative multiple pregnancy rate	17.6%	18.4%	0.667
OHSS rate	4.9%	2.0%	0.001
Cumulative ectopic pregnancy rate	2.9%	4.0%	0.26

AFC: antral follicle count; bFSH: basal follicle stimulating hormone; bLH: basal luteinizing hormone; bE2: basal estrogen; OHSS: ovarian hyperstimulation syndrome.

### COH protocols

GnRH-agonist long-protocol: A short-acting GnRH-a (Triptorelin, Ferring AG, Germany) was administrated daily in the mid luteal phase of the preceding cycle. 14 days later, follicular ultrasonography, serum LH, FSH and E2 were examined and 150–300IU recombination follicle stimulating hormone (r-FSH, Gonal-F, Merck Serono, Switzerland) was initiated daily when FSH and LH < 5 IU/L and E2 < 50 pg/ml. GnRH-a was continued until trigger. During the stimulation, according to ovarian response evaluated by transvaginal ultrasonography and serum hormone level, dose of r-FSH was adjusted as needed, human menopausal gonadotropin (HMG) or recombinant luteinizing hormone(r-LH) or growth hormone (GH) was added as needed.

GnRH-antagonist protocol: 150–300IU r-FSH was initiated on day 2 or 3 of the menstrual cycle until trigger. The dosage of r-FSH was adjusted and HMG or r-LH or GH was added according to the ovarian response evaluated by transvaginal ultrasonography and serum hormone level. 0.25 mg GnRH-A (Cetrorelix; Merck Serono, France) was used daily until trigger when the leading follicles reached a mean diameter of 14 mm.

For both protocols, if three follicles reached a mean diameter of 17 mm or two follicles reached a mean diameter of 18 mm, r-HCG (Ovidrel, Serono, Italy) was administered subcutaneously. Oocyte retrieval was performed 36 h after HCG injection by transvaginal ultrasound-guided single-lumen needle aspiration. Luteal phase support was initiated on day 1 after oocyte retrieval. Fresh embryo transplantation was carried out 72 h after oocyte retrieval. Fresh cycles were canceled if patients had endometrial thickness <7 mm, high risk of Ovarian hyperstimulation syndrome (OHSS) (E2 ≥ 5000 pg/ml on the trigger day, the number of oocytes obtained ≥20), no available embryos or other personal reasons. For those patients without fresh embryo transplantation or without ongoing pregnancy after fresh embryo transplantation, if excess frozen embryos were available and pregnancy test was negative, the frozen embryo transplantation (FET) was performed until no embryos remained or ongoing pregnancy was achieved. Clinical pregnancy was defined as a gestational sac observed by vaginal ultrasound. Ongoing pregnancy was defined as a pregnancy continuing 12 weeks without miscarriage.

### Structure of the model

**Figure 1:**
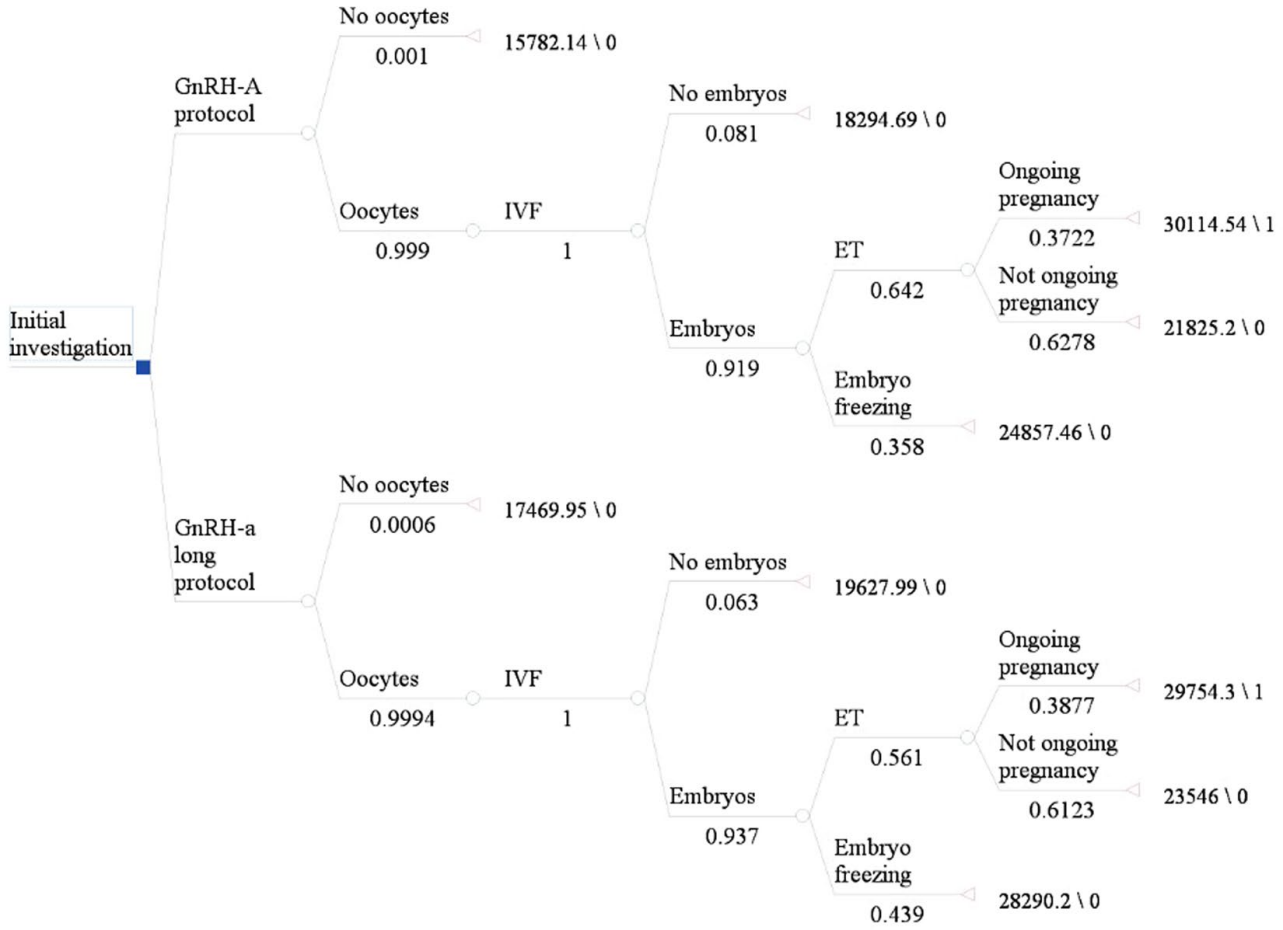
Decision tree model for fresh embryo transplantation. Figure 1 shows the process of fresh embryo transplantation. The number under each node in the figure is the correlation probability of this step.

**Figure 2:**
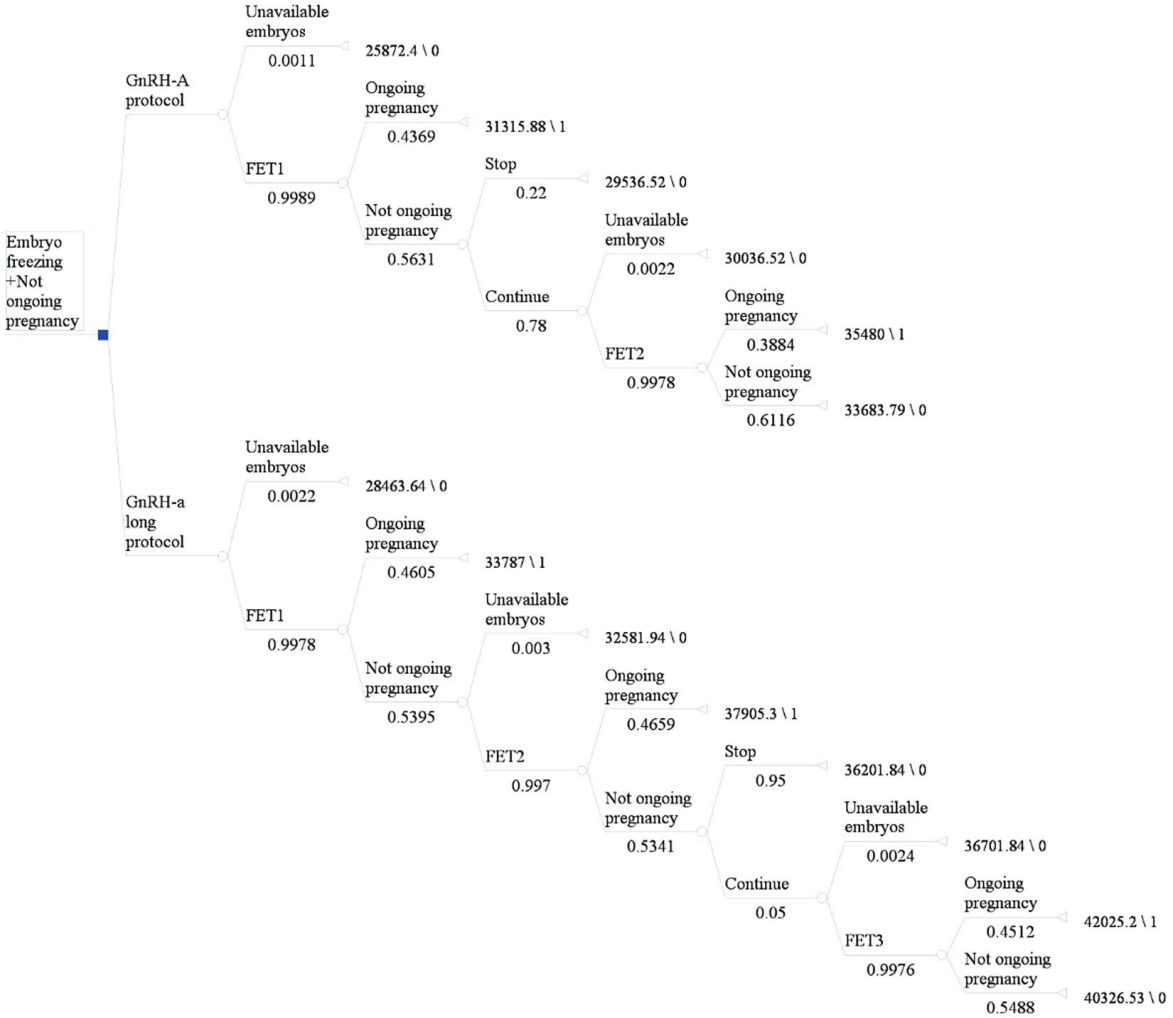
Decision tree model for frozen embryo transplantation. Figure 2 shows the frozen embryo transplantation process after fresh embryo transplantation. The number under each node in the figure is the correlation probability of this step.

We constructed a decision tree model to analyze the cost-effectiveness of the GnRH-agonist long-protocol and GnRH-antagonist protocol in the fresh embryo transplantation and frozen embryo transplantation cycle (Fig. 1 and Fig. 2). Each route in the diagram represents possible steps in IVF. Each intersection is followed by a possible situation. Since the cumulative ongoing pregnancy rate was calculated by following utilization of all fresh and frozen embryos after the first IVF cycle, the number of transplants was based on available embryos. The terminal nodes of the model were: “No oocytes”, “No embryos”, “Ongoing pregnancy”, “No ongoing pregnancy”. Since each patient may have different situations after entering the cycle, we made relevant assumptions for the model for the convenience of calculation as shown in Table 2.

**Table 2:** Assumptions in the model.

Assumptions
1.r-LH and GH were added according to the development of follicles, not all patients used it, so their costs were not included in the total cost.
2. In GnRH-agonist long-protocol, there were an average of 5 blood tests and follicular ultrasonography examinations, while in the GnRH-antagonist protocol, there were 4 blood tests and follicular ultrasonography examinations.
3. Artificial cycle drug was unified as estradiol valerate tablets, with an average of 4 follicular ultrasound tests. There were 4 follicular ultrasound tests on average in the natural cycle.
4. The corpus luteum support drugs were unified as estradiol valerate + dydrogesterone tablets + progesterone vaginal sustained release gel in fresh cycle and estradiol valerate + dydrogesterone tablets + progesterone capsules in frozen cycle.
5. The cost of lost work were calculated based on the per capita disposable income of the three-year residents in 2015–2017.
6. The transportation fee was calculated according to the second-class fare of the trains from various cities in Zhejiang Province to Hangzhou.
7. The bed fee during hospitalization was calculated at a rate of ¥40 per day for a three-person room.
8. The number of frozen embryo transfer cycles is calculated based on the number of frozen embryos and the number of transplanted embryos.

### Transition probabilities

The transition probabilities for the various health states in this decision tree model were derived from the clinical data of included infertility patients. The transition probability of each step is shown in Fig. 1 and Fig. 2.

### Cost analysis

This study was performed from a patient’s perspective. Both direct and indirect costs were included in the analysis. Direct medical costs included drug, ultrasound, laboratory, surgery and care costs. Medications included those used in ovulation stimulation trigger and luteal support. The cost of the drug was equal to the unit cost of the drug multiplied by the total amount used. When calculating the cost of IVF, it is prudent to include the treatment costs for complications, primarily OHSS. Therefore, the cost of treatment for OHSS was also calculated in the total cost of the patient. In this study, transportation costs were included as direct non-medical expenses. These were calculated as the average fare from each city in Zhejiang province to Hangzhou. Indirect costs included the cost of lost work. The cost of lost work was calculated according to the per capita disposable income. Intangible economic burden generally refers to the decline in the quality of life or other costs caused by illness in this case, IVF, and also included other costs not reflected in the direct and indirect costs. Thus, considering the difficulty in calculating these intangible costs, such costs were not included in this study. Cycle costs were relatively stable during the study period, so discounting is not considered. The specific costs are shown in Table 3.

**Table 3:** The cost involved in IVF.

Drugs	Costs ($)	Operation	Costs ($)	Non-medical expenses	Costs ($)
Triptorelin(0.1 mg)	16.33	**Oocyte retrieval**	305.81	**Single transportation fee**	11.61
Gonal-F(450IU)	230.26	**Anesthetic Fee**	96.02	**The daily cost of lost work**	10.02
HMG(75IU)	3.18	**IVF**	305.81	**Daily nursing expenses during hospitalization**	3.67
Cetrorelix(0.25 mg)	52.72	**Embryo transplantation**	244.65	**Bed fee per day during hospitalization**	6.12
Ovidrel(6500IU)	29.5	**Embryo cryopreservation**	611.62		
Estradiol valerate tablets(tablet)	0.26	**Embryos thawing**	76.45		
Dydrogesterone tablets(tablet)	0.83	**Follicular ultrasonography**	6.42		
Progesterone vaginal sustained release gel	11.47	**Serum FSH,LH,E2,Ptest**	4.89		
Progesterone capsules (capsule)	0.43	**Serum HCG**	6.12		

### Statistical analyses

The statistical software package SPSS22 was used for data analysis. When the measurement data matched the homogeneity of variance, the independent sample t test was used; when the data didn’t match the homogeneity of variance, the Mann-Whitney test was used. The chi-square test or Fisher’s exact probability method were used for the comparison of the rates, and P < 0.05 was considered statistically significant. Cost-effectiveness analysis and incremental cost-effectiveness analysis were used to evaluate the costs and effects between the two protocols. Sensitivity analyses were then performed to assess the stability of the model.

### Ethical approval

The study was approved by the Ethics Committee of Women’s Hospital School of Medicine Zhejiang University. All patients meeting the inclusion criteria signed the informed consent. And this study complied with declaration of Helsinki/relevant guidelines for the study on humans.

## Results

About 2259 infertility patients met inclusion criteria (1638 in GnRH-agonist long-protocol and 621 in GnRH-antagonist protocol). There were no differences in patient ages, BMI, duration of infertility, cycle day 3 LH levels, cycle day 3 E2 levels and AFC. The duration of gonadotrophin stimulation and dose of gonadotropin used, number of oocytes obtained and number of available embryos in GnRH-agonist long-protocol were higher than that in GnRH-antagonist protocol. The clinical pregnancy rate and ongoing pregnancy rate for GnRH-agonist long-protocol and GnRH-antagonist protocol in the fresh embryo transplantation cycle were not different (47.26% vs. 49.44%, P = 0.487; 38.77% vs. 37.22%, P = 0.613). When all available embryos had been utilized, the rate of cumulative ongoing pregnancy per start cycle was 60.65% for GnRH-antagonist protocol and 71.6% for GnRH-agonist long-protocol (P < 0.01). The OHSS rate was lower in GnRH-antagonist protocol (2.0% vs 4.9%, P = 0.001). The cumulative multiple pregnancy (17.6% vs 18.4%, P = 0.667) rate and the cumulative ectopic pregnancy rate (2.9% vs 4.0%, P = 0.26) in GnRH-agonist long-protocol was not different from that of GnRH-antagonist protocol. Data on patients’ demographic and infertility treatment-related characteristics are represented in Table 1.

In fresh embryo cycles, there were less amount of gonadotropin and few days of OHSS for GnRH-antagonist protocols. Therefore, the total cost was lower in GnRH-antagonist protocols (average cost $16970.85) than in GnRH-agonist long-protocol (average cost $19902.24) in fresh embryo cycles. In fresh embryo transfer cycles, there was no statistical difference in the ongoing pregnancy rate between the two protocols, therefore, the minimum cost method was used for analysis. When the cumulative ongoing pregnancy rate was taken into account in the long protocol group, the number of frozen embryo transfer cycle was higher, leading to a higher cumulative ongoing pregnancy rate, and consequently a higher cost. Using cost-effectiveness analysis, the mean costs per ongoing pregnancy were estimated at $8176.76 and at $7595.28 with GnRH-antagonist protocols and GnRH-agonist long-protocols, respectively. The incremental cost-effectiveness ratio (ICER) is an evaluation index commonly used in economics research. It refers to the ratio of cost difference and effect difference between different protocols. In this study, the ICER for GnRH-agonist long-protocol versus GnRH-antagonist protocol was estimated at $4375.95 for one more ongoing pregnancy (Table 4).

**Table 4:** Cost-Effectiveness analysis of GnRH-antagonist protocol and GnRH-agonist long-protocol.

Protocols	Costs ($)	Effective	C/E ($)	ICER ($)
GnRH-antagonist protocol	4958.79	0.6065	8176.76	—
GnRH-agonist long-protocol	5438.12	0.716	7595.28	4375.95

Sensitivity analysis is to change the value of some parameters and analyze the degree of influence on the results. The cost of ovarian stimulation protocols account for the majority of the total costs^10^. In this study, the cost of drugs accounted for the largest proportion of the total cost of ovulation stimulation protocols. The reliability of the results was assessed by sensitivity analysis with drug cost fluctuation. The results of sensitivity analysis were consistent with the results of cost-effectiveness analysis, the cost per ongoing pregnancy in the GnRH-agonist long-protocol was lower than that in the GnRH-antagonist protocol, which means the results are stable.

## Discussion

In this study, we used an economics research method to evaluate the cost-effectiveness of the GnRH-agonist long-protocol and the GnRH-antagonist protocol for IVF. We found that in the fresh embryo transplantation cycle, there was no significant difference in the ongoing pregnancy rate between the GnRH-agonist long-protocol and the GnRH-antagonist protocol, while the cost in the GnRH-antagonist protocol was lower, so GnRH-antagonist protocol has advantage according to the principle of cost-minimization analysis of pharmacoeconomics. When considering the cumulative ongoing pregnancy rate after each ovarian stimulation, the cumulative ongoing pregnancy rate and cost in the GnRH-agonist long-protocol were higher than the GnRH-antagonist protocol. Using the cost-effectiveness analysis method, it was found that the average cost of each ongoing pregnancy in the GnRH-agonist long-protocol was lower than the GnRH-antagonist protocol. Therefore, the GnRH-agonist long-protocol is more cost-effective.

Studies have shown that one of the primary reasons for dropout from infertility treatment is economic burdens^12–14^. China’s medical system does not provide insurance coverage for infertility diagnosis and treatment^15^. It can be an enormous economic burden to patients seeking ART. Therefore, no matter from the perspective of patients, or from the perspective of medical resource allocation, it is necessary to carry out economic analysis on IVF and consider the cost and effect of each step. Although GnRH-agonist long-protocols and GnRH-antagonist protocols have been widely used in IVF, there is still an ongoing debate about the results of the two protocols. Orvieto^16^ and Grow^17^ found the GnRH-agonist protocol has a superiority over the GnRH-antagonist protocol in live birth rate. Some studies also found no significant difference in the rates of live births or ongoing pregnancies between the two protocols^18,19^. However, these studies are only from the perspective of clinical results, not from the perspective of economics. In our research, we hope to be able to focus more on evaluating the cost-effectiveness of both protocols, not just the clinical outcomes, which are just a link in the evaluation. After consulting the literature, we found that there have been little economic studies on the two different ovarian stimulation protocols used in IVF. Wei Pan^20^ *et al*. conducted a retrospective analysis of the cost-effectiveness of GnRH-a protocols, GnRH-ant protocols and GnRH-a ultra-long protocols. They used the live birth rate as one outcome of the study. However, it is difficult to calculate the cost throughout pregnancy. In order to ensure that the results are more reliable, we used the ongoing pregnancy rate as the end point of this economic study.

The cost of IVF treatment for infertility (the cost per ongoing pregnancy) in this study was higher than the average hospitalization cost for 30 diseases in 2018 according to the national bureau of statistics^21^. IVF is a complex process involving ovarian stimulation, ovum retrieval, fertilization, embryo transfer and other processes. In addition to the cost of these processes, the total cycle costs of IVF should also include the transportation costs, lost wages and the cost of treating OHSS. However, it is difficult to accurately assess the transportation costs and lost wages and these indirect cost consisted a small percentage of the total costs, many studies did not include them in the total cycle cost analysis^22^. But, from the perspective of patients, these costs are indirect medically, yet direct economically to patients, so they are also included in this study. OHSS is a serious complication of IVF, and the treatment is expensive, which directly affects the total cycle costs. Studies have shown that the incidence of OHSS in the GnRH-antagonist protocol is lower than that in the GnRH-agonist long-protocol^18,23,24^. Accordingly, the cost of treating OHSS in the GnRH-antagonist protocol is lower, resulting in reduction in the total cycle cost. This may be one of the reasons why the cost in GnRH-antagonist protocol is lower than the GnRH-agonist long-protocol in the fresh embryo transfer cycle.

Economic analysis of IVF is still challenging, and there is no unified view on the result indicators of the analysis. Existing economic studies often used ongoing pregnancy, live birth rates or quality-adjusted life years (QALYs) as result of the study. However, it is worth considering that QALYs of both husband and wife or child is used when taking QALYs as a result of IVF^25^. Toftager^26^ *et al*. found that quality of life and psychosocial and physical well-being of patients used the GnRH-antagonist protocol was better than that used the GnRH-agonist protocol. But these are hard to quantify in terms of costs. And it’s difficult to calculate the impact on families and society of obtaining a healthy baby by IVF. Considering the different incidence of related complications during pregnancy, the treatment costs and nursing costs vary greatly. Therefore, in this study, we used the cumulative ongoing pregnancy rate as the effect of the study.

There are many economic analysis methods, but among the existing studies on economics in ART, 84% are cost-effectiveness analysis and 48% are model-based studies^11^. Economic model carried out before a trial is particularly useful in reducing unnecessary waste of research resources in evaluating techniques and interventions and improving the quality and efficiency of the research^27^. In our study, we developed a decision-tree model to evaluate the cost-effectiveness of the GnRH-agonist long-protocol and GnRH-antagonist protocol. The relevant transfer probability in the model was calculated using the data of infertile patients in the reproductive center of our hospital. In addition, we have made some reasonable assumptions for analysis, as shown in Table 2.

This is a retrospective study on the data available from infertility diagnosis and treatment in our reproductive center. The relevant probability and costs in the model are calculated based on the data of our single center. These results may differ from those of other centers, but can provide some guidance for patients and clinicians. In the future, large samples, multi-center prospective randomized controlled trials are needed to more thoroughly explore more economical and effective treatment protocols.

In conclusion, if fresh embryo transfers are considered, the pregnancy outcomes between GnRH-agonist long protocols and GnRH-antagonist protocols are similar, but GnRH-antagonist protocols have lower cost. Therefore, in the fresh embryo transfer cycle, the GnRH-antagonist protocol has economic advantage and is worth recommending. However, GnRH-agonist long-protocol have higher success rates and higher costs when cumulative ongoing pregnancy rates are taken into account. The cost per ongoing pregnancy in the GnRH-agonist long-protocol cycles was lower than that in the GnRH-antagonist protocol cycles. Thus, cost-effectiveness analysis shows that the GnRH-agonist long-protocol is more cost-effective than the GnRH-antagonist protocol and may represent a cost-effective option from the perspective of patients. However, further large sample sizes and multi-center randomized controlled trials are needed.

## References

[1] Mascarenhas Maya N., Flaxman Seth R., Boerma Ties, Vanderpoel Sheryl, Stevens Gretchen A. (2012). National, Regional, and Global Trends in Infertility Prevalence Since 1990: A Systematic Analysis of 277 Health Surveys. PLoS Medicine.

[2] European Society of Human Reproduction and Embryology. *More than 8 million babies born from IVF since the world’s first in 1978*., (2018).

[3] Polyzos NP (2018). Cumulative live birth rates according to the number of oocytes retrieved after the first ovarian stimulation for *in vitro* fertilization/intracytoplasmic sperm injection: a multicenter multinational analysis including approximately 15,000 women. Fertility and sterility.

[4] Coccia ME, Comparetto C, Bracco GL, Scarselli G (2004). GnRH antagonists. European journal of obstetrics, gynecology, and reproductive biology.

[5] Fleming RC (1986). J. R. Induction of multiple follicular growth in normally menstruating women with endogenous gonadotropin suppression. Fertility and sterility.

[6] Huirne JA, Homburg R, Lambalk CB (2007). Are GnRH antagonists comparable to agonists for use in IVF?. Human reproduction (Oxford, England).

[7] Diedrich K (1994). Suppression of the endogenous luteinizing hormone surge by the gonadotrophin-releasing hormone antagonist Cetrorelix during ovarian stimulation. Human reproduction (Oxford, England).

[8] Hall JE (1988). Evidence of differential control of FSH and LH secretion by gonadotropin-releasing hormone (GnRH) from the use of a GnRH antagonist. The Journal of clinical endocrinology and metabolism.

[9] Ombelet W, Campo R (2007). Affordable IVF for developing countries. Reproductive biomedicine online.

[10] Bouwmans CA (2008). A detailed cost analysis of *in vitro* fertilization and intracytoplasmic sperm injection treatment. Fertility and sterility.

[11] Moolenaar LM (2013). Economic evaluation studies in reproductive medicine: a systematic review of methodologic quality. Fertility and sterility.

[12] Domar AD, Smith K, Conboy L, Iannone M, Alper M (2010). A prospective investigation into the reasons why insured United States patients drop out of *in vitro* fertilization treatment. Fertility and sterility.

[13] Mourad SM (2010). Determinants of patients’ experiences and satisfaction with fertility care. Fertility and sterility.

[14] van Dongen AJ, Verhagen TE, Dumoulin JC, Land JA, Evers JL (2010). Reasons for dropping out from a waiting list for *in vitro* fertilization. Fertility and sterility.

[15] Zheng XY, Disease QY (2012). burden of infertility in China. Chin J Public Health.

[16] Orvieto R, Patrizio P (2013). GnRH agonist versus GnRH antagonist in ovarian stimulation: an ongoing debate. Reproductive biomedicine online.

[17] Grow D (2014). GnRH agonist and GnRH antagonist protocols: comparison of outcomes among good-prognosis patients using national surveillance data. Reproductive biomedicine online.

[18] Wang RL, Lin SR, Wang Y, Qian WP, Zhou L (2017). Comparisons of GnRH antagonist protocol versus GnRH agonist long protocol in patients with normal ovarian reserve: A systematic review and meta-analysis. PloS one.

[19] Al-Inany HG (2016). Gonadotrophin-releasing hormone antagonists for assisted reproductive technology. The Cochrane database of systematic reviews.

[20] Pan W (2019). Decision analysis about the cost-effectiveness of different *in vitro* fertilization-embryo transfer protocol under considering governments, hospitals, and patient. Medicine.

[21] National Health Commission. China health statistics yearbook 2018 115 (China union medical university press (2018).

[22] Collins J (2002). An international survey of the health economics of IVF and ICSI. Human reproduction update.

[23] Lambalk CB (2017). GnRH antagonist versus long agonist protocols in IVF: a systematic review and meta-analysis accounting for patient type. Human reproduction update.

[24] Chappell N, Gibbons WE (2017). The use of gonadotropin-releasing hormone antagonist post-ovulation trigger in ovarian hyperstimulation syndrome. Clinical and experimental reproductive medicine.

[25] ESHRE Capri Workshop Group. (2015). Economic aspects of infertility care: a challenge for researchers and clinicians. Human reproduction (Oxford, England).

[26] Toftager M (2018). Quality of life and psychosocial and physical well-being among 1,023 women during their first assisted reproductive technology treatment: secondary outcome to a randomized controlled trial comparing gonadotropin-releasing hormone (GnRH) antagonist and GnRH agonist protocols. Fertility and sterility.

[27] Torgerson DJ, Byford S (2002). Economic modelling before clinical trials. BMJ (Clinical research ed..

